# Vitamin D Status among Pulmonary TB Patients and Non-TB Controls: A Cross-Sectional Study from Mwanza, Tanzania

**DOI:** 10.1371/journal.pone.0081142

**Published:** 2013-12-06

**Authors:** Henrik Friis, Nyagosya Range, John Changalucha, George PrayGod, Kidola Jeremiah, Daniel Faurholt-Jepsen, Henrik Krarup, Christian Mølgaard, Åse Bengaard Andersen

**Affiliations:** 1 Department of Nutrition, Sports and Exercise, University of Copenhagen, Frederiksberg, Denmark; 2 National Institute for Medical Research, Muhimbili Medical Research Centre, Dar es Salaam, Tanzania; 3 National Institute for Medical Research, Mwanza Medical Research Centre, NIMR, Mwanza, Tanzania; 4 Department of Clinical Biochemistry, Aalborg University Hospital, Aalborg, Denmark; 5 Department of Infectious Diseases, Odense University Hospital, Odense, Denmark; McGill University, Canada

## Abstract

**Background:**

Little is known about vitamin D status in low-income populations burdened with infectious diseases. Hence, there is a need for data on correlates of serum 25-hydroxy vitamin D (S-25(OH)D) and its validity during infections.

**Objective:**

To assess the role of pulmonary TB (PTB) and HIV as correlates of S-25(OH)D.

**Design:**

Age-sex-matched cross-sectional study among PTB patients and non-TB controls.

**Methods:**

PTB patients were categorized as sputum negative (PTB−) and positive (PTB+) by culture. Non-TB controls were randomly selected among age-sex-matched neighbours to PTB+ patients. Height, weight, arm circumference and triceps skinfold were measured, and body mass index (BMI), arm fat (AFA) and muscle area (AMA) computed. HIV status, and S-25(OH)D, C-reactive protein (S-CRP) and α_1_-acid glycoprotein (S-AGP) were determined. Linear regression analysis with controls and PTB patients combined was used to identify correlates of S-25(OH)D.

**Results:**

S-25(OH)D data were available on 97.8% (1570) of 1605 participants. Mean (SD) S-25(OH)D was 84.4 (25.6) nmol/L with 39.6% <75 nmol/L among 347 non-TB controls. Time of recruitment, sex, PTB and HIV, and elevated S-AGP were correlates of S-25(OH)D. S-25(OH)D was 24.8 (95% CI 18.6;30.9) nmol/L higher in PTB compared to controls among females, but only 9.8 (95% CI:4.5;15.2) nmol/L among males (interaction p<0.0001). Females had 13.8 (95% CI:8.2;21.9) nmol/L lower S-25(OH)D than males, and HIV infected individuals had 8.5 (95% CI:4.9;12.1) higher S-25(OH)D compared to uninfected. Elevated S-AGP was a positive correlate of S-25(OH)D. Low BMI was associated with S-25(OH)D, but not with infections or S-AGP in the model.

**Conclusion:**

While S-25(OH)D may decline transiently during a mild acute phase response, it may increase if the acute phase response leads to loss of fat. The validity of S-25(OH)D as a marker of vitamin D status may be affected by infections.

## Introduction

Although vitamin D is mainly synthesized in the skin during sun exposure, hypovitaminosis D has been shown to be common in low-income countries, including those in equatorial Africa [1]–[3]. Since vitamin D is an immunoregulatory hormone and a possible determinant of infectious diseases [4], inadequate vitamin D status may be a public health problem.

There is evidence to suggest that hypovitaminosis D increases the risk of tuberculosis (TB) [5]. Vitamin D binds to nuclear receptors in macrophages, leading to an oxidative burst which is important for the intracellular antimycobacterial activity [6], and vitamin D metabolites upregulate nitric oxide synthase which serves to suppress mycobacterial growth [7]. Yet, the epidemiological evidence is not convincing. No data from trials on the effect of vitamin D on risk of TB disease have been reported [8], and the results from case-control studies are inconsistent [3], [5], [9]. A problem with case-control studies is that vitamin D status is assessed when TB is diagnosed, at which time pathogenic disease processes are already flourishing. Associations between low serum concentrations of 25-hydroxy-cholecalciferol (serum 25(OH)D) and TB reported from many case-control studies are interpreted as reflecting that low vitamin D status increases the risk of TB. Nevertheless, this association could be explained by reverse causality, if TB itself impairs vitamin D status or if the acute phase response precipitated by TB impairs the validity of serum 25(OH)D as a marker of vitamin D status. Indeed, a couple of studies have demonstrated that serum 25(OH)D decline following during surgery [10], [11].

We have previously reported hypovitaminosis D among 655 pulmonary TB (PTB) patients in Mwanza, Tanzania, which was not explained by the acute phase response [1], but lack of appropriate non-TB controls in that study was a limitation. Therefore, as part of a larger nutrition study, we obtained cross-sectional data on serum 25(OH)D among PTB patients and age-sex-matched neighbourhood controls, with an aim to assess vitamin D status and the role of PTB and HIV as correlates of serum 25(OH)D.

## Methodology

### Ethics Statement

Ethical permission was obtained from the Medical Research Coordinating committee of the National Institute for Medical Research in Tanzania, and consultative approval was given by The Danish Central Medical Ethics Committee. Written and oral information was presented to all eligible participants by the health staff before written informed consent was obtained. Written consent was obtained from parents/legal guardians of any participants under 18 years of age.

### Study Setting and Design

A cross-sectional study conducted from April 2006 to March 2009 in Mwanza City, Tanzania, among PTB patients recruited for a large nutrition intervention study and non-TB controls. Mwanza City is at the shores of Lake Victoria, at latitude 2.28 S, longitude 32.55 E, and an altitude of 1140 m. The annual rainfall is 700–1000 mm, with long rains from February to April and short rains in November and January. The mean number of daily sunshine hours ranges from 7.0 to 9.0 per day, lowest in October to April with 7.0–7.4 h/d and highest in June and July with 9.0 h/d [1]. The population is generally unveiled, with faces and often arms exposed to the sun. The harvest is from May to July, and the staple foods are maize, cassava, sweet potato, rice, and millet. Fish is the most common animal-source food.

### Recruitment and Management of TB Patients

The PTB patients were recruited at the four TB clinics under the TB treatment services, coordinated by the National Tuberculosis and Leprosy Programme. Smear-positive (PTB+) or –negative (PTB−) patients were enrolled in the study after giving informed consent if they were residents of Mwanza city, to allow follow-up. Patients with extra-pulmonary TB, pregnancy, age under 15 years, or terminal illness were excluded. The diagnosis of TB followed the World Health Organization (WHO) guidelines [12] using the Ziehl–Neelsen staining technique [13]. Briefly, all TB suspects were asked to bring three sputum samples, ie spot-morning-spot samples, for microscopy, and were asked to do a chest X-ray as appropriate. Patients were considered to be smear-positive, if two samples tested positive or one sample tested positive and a chest X-ray was suggestive of TB, and to be smear-negative, if all the samples were negative, but chest X-ray was suggestive of TB, and there was non-response to a course of broad-spectrum antibiotics, eg amoxicillin. After diagnosis all patients were started on a standardized TB treatment for 6–8 months based on existing national guidelines [14], [15]. The management of HIV infection was done based on national guidelines at the time of the study [16]. Patients were supposed to start antiretroviral therapy (ART) if they had CD4 count of <200 cells/µl, those with WHO stage 4 illness and/or CD4 count of 200–350 cells/µl were supposed to start ART after completion of two months of TB treatment. Patients who developed TB after starting ART continued with ART throughout TB treatment.

### Recruitment of Non-TB Controls

Among PTB patients recruited for the nutrition intervention trials (clinicaltrials.gov, NCT0031129) it was planned to designate up to 400 consecutive smear-positive participants as index cases for selection of age-sex-matched neighbourhood non-TB controls. Mwanza City is divided into wards, streets and communal cells. Each cell has 10–20 households, and is headed by a ten cell-leader. Each of the index patients who were eventually recruited was asked to provide his/her residential address and the name of his/her ten-cell leader. Using this information, the study team requested the ten-cell leader to provide the complete list of individuals in his/her jurisdiction meeting the age and sex recruitment criteria. Of these, one was randomly selected using a lottery method and invited to participate in the study as a non-TB control if meeting the following criteria: no history of previous or TB exposure, active TB or TB treatment, no evidence of current active TB (cough, intermittent fevers, and excessive night sweating in the past two weeks and unexplained weight loss in the past month), same sex as index case, aged 15 years or above and age difference from index case was not more than five years, had lived in the same street as index case for at least three months, not pregnant, and consenting to participate in the study. Persons who were terminally ill were not invited. If the selected individual declined to participate, then another from the list was randomly selected.

### Data Collection

For the purpose of the study, all PTB patients provided an additional sputum sample for culture at the Zonal TB Reference Laboratory, and were subsequently categorized as sputum culture-positive (PTB+) or culture-negative (PTB−). All PTB patients and controls had data on demography, smoking, and alcohol intake collected using questionnaires, while data on antiretroviral treatment (ART) were retrieved from antiretroviral-use databases in ART clinics. Weight, height, mid-upper arm circumference (MUAC) and triceps skin fold thickness (TSFT) were determined. From weight and height, body mass index (BMI) was calculated using the formula: weight (kg)/(height (m))2. From MUAC and TSFT, arm muscle area and arm fat area were calculated using the formulae: arm muscle area = [MUAC−(TSFT×π)]^2^/(4×π) and arm fat area = [MUAC^2^/(4×π)] – arm muscle area, as measures of lean and fat mass [17]. Between 8 am and 12 noon, as soon as possible and preferably before start of TB treatment, blood was collected in a 10 ml plain vacutainer tube for HIV testing and a 5 ml EDTA vacutainer tube for CD4 count. HIV status was determined using Capillus HIV-1/HIV-2 (Trinity Biotech Plc., Wicklow, Ireland) and Determine HIV-1/HIV-2 (Inverness Medical Innovations, Inc., Delaware, U.S.A.) tests in parallel [18]. HIV infection was diagnosed if both tests gave a positive result and HIV negative diagnosis was made if both tests produced a negative result. Indeterminate results were resolved using ELISA– Organon Uniform II (Organon Teknia Ltd, Boxtel, Netherlands) [19]. CD4 count was determined as cells/µl using a Partec Cyflow Counter (Partec GmbH, *Münster*, Germany). Serum samples were kept frozen at −80°C at NIMR lab after collection and were transported to Denmark on dry ice and kept at −80°C until analysis, after a maximum of 3 years storage, of serum 25(OH)D were conducted at Aalborg Hospital, FBE Clinical Biochemistry South, using a fully automated direct competitive chemiluminescent immunoassay (LIAISON 25-OH Vitamin D TOTAL, DiaSorin Inc, Stillwater, Minnesota, USA) [20]. Within-run and total precision were 2.9–5.5% and 6.3–12.9%, respectively. Serum 25(OH)D <50 nmol/L was used to define vitamin D deficiency and <75 nmol/L to define hypovitaminosis D [21]. To be able to adjust for the acute phase response, serum α_1_-acid glycoprotein (AGP) was determined with a standard Alpha_1_-Acid Glycoprotein Kit using Beckman Coulter Image® Immunochemistry Systems (Beckman Coulter, Galway, Ireland) and C-reactive protein (hsCRP) was determined with Tina-quant C-Reactive Protein Gen.3 (CRPL3) on a Roche COBAS 6000 instrument (Roche Diagnostics GmbH, Mannheim, Germany).

### Statistical Analysis

Normal probability plots were used to assess the distribution of continuous variables. Chi-square test was used to test for differences in proportions. The two-sample t test or oneway ANOVA were used to test for differences in means between two or more groups, respectively. The analysis was done with controls and PTB combined. Linear regression analysis was used to identify correlates of serum 25(OH)D as a continuous variable, and to test for interactions with PTB. The variables assessed were age, sex, anthropometry, PTB and HIV status, and elevated serum CRP (>2 mg/L) or AGP (>1.0 mg/L), with adjustment for season through variables corresponding to quarter and year of recruitment, with all variables initially in the model. Models are presented with and without adjustment for elevated serum AGP using dummy variables with values below 1.0 mg/L as reference category. If interaction with PTB status was identified, then the association was expressed separately for each. Normal and residual-vs.-fitted plots were used to assess normality and homoscedasticity of residuals. Stata version 11.0 (StataCorp, Texas, USA) was used for all analyses.

## Results

Of 1605 individuals included in the study, 1250 (77.9%) were PTB patients and 355 (22.1%) controls. Of the PTB patients, 427 (34.2%) were PTB− and 823 (65.8%) PTB+. HIV prevalence was higher among PTB patients compared to controls (50.4 vs 9.9%, p<0.001), and higher among PTB− compared to PTB+ patients (64.4 vs 43.1%, p<0.001). Of 636 HIV infected, 79 (12.2%) were on ART. Data on serum 25(OH)D were available on 1569 (97.8%) of the 1605 participants.

**Figure 1:**
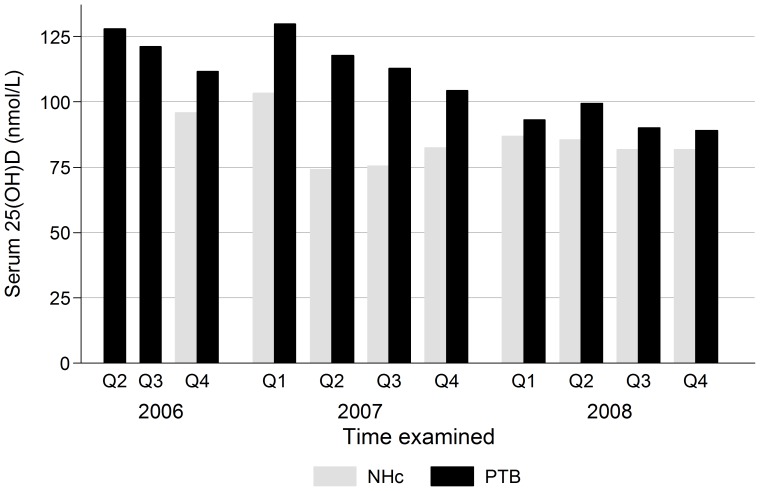
Mean serum 25(OH)D among 355 neighbourhood controls (NHc) and 1250 pulmonary tuberculosis (PTB) patients at the year and quarter (Q) of examination. Two cases from first quarter of 2006 and seven from 2009 not shown.

Among 347 non-TB controls, mean (SD) serum 25(OH)D was 84.4 (25.6) nmol/L, with 39.6% (137) having hypovitaminosis D (<75 nmol/L), including 4.3% (15) with vitamin D deficiency (<50 nmol/L). Serum 25(OH)D was 26.4 (95% CI: 22.4; 30.5) nmol/L higher among the 1223 PTB patients, with mean (SD) of 110.9 (35.7) nmol/L, and 15.0% (184) had hypovitaminosis D, including 2.5% (31) with vitamin D deficiency. Among PTB patients, the mean (range) delay of blood sampling for serum 25(OH)D determination was 2.0 (0–14) days after initiation of TB treatment. Mean serum 25(OH)D declined with time after treatment start, as it was 113.1 nmol/L among the 540 (44.9%) examined at the day of treatment, and 110.0 nmol/L among the 521 (43.3%) examined within 1–5 days and 104.8 nmol/L among the 143 (5.7%) examined within 6–14 days (p = 0.04, linear trend p = 0.01). The PTB patients were recruited from April 2006 to November 2008, and controls from October 2006 to January 2009. For both groups, serum 25(OH)D varied considerably between and within years (**Figure 1** **)**. Among PTB patients, levels were higher in 2006 and 2007 compared to 2008, and in the first or second quarter. A less distinct pattern was seen for controls, recruited with up to two months delay.

Serum 25(OH)D by sex, age, BMI, TB and HIV and ART status, and elevated serum acute phase reactants are shown in **Table 1**, for TB patients and controls combined. As seen, low BMI was associated with higher serum 25(OH)D. Similar patterns were seen for AMA and AFA (data not shown). There was no difference in serum 25(OH)D between PTB− and PTB+ patients (111.8 vs 110.4 nmol/L, p = 0.53), but PTB patients together had 26.4 (95% CI: 22.4; 30.5) nmol/L higher serum 25(OH)D than controls. The difference by TB status was also determined among the 345 pairs of PTB+ index cases and controls only, and serum 25(OH)D was found to be 21.8 (95% CI: 17.2; 26.4) nmol/L higher in the PTB+ patients (data not shown). Similarly, ART-naïve HIV infected had higher serum 25(OH)D than HIV uninfected, while HIV infected on ART was in between. Elevated serum CRP or AGP were also positively associated with serum 25(OH)D.

**Table 1:** Serum 25(OH)D in 1250 pulmonary TB patients and 355 neighbourhood controls by categories of sex, age, body mass index, pulmonary TB and HIV status and elevated acute phase reactants^1^.

	% (n)	Mean	95% CI	P
Sex				0.18
Females	41.9 (657)	103.6	100.9; 106.3	
Males	58.1 (912)	106.0	104.7; 108.8	
Age (y)				<0.001
<25	22.2 (349)	99.0	95.5; 102.5	
25–35	33.6 (527)	106.3	103.4; 109.2	
35–45	24.0 (377)	107.4	103.5; 111.4	
45–55	12.2 (192)	110.5	105.3; 115.6	
55+	7.9 (124)	101.0	95.3; 106.8	
Pulmonary TB status^1^				<0.001
Non-TB control	22.1 (346)	84.4	81.7; 87.1	
PTB−	26.4 (414)	111.8	108.2; 115.4	
PTB+	51.6 (809)	110.4	108.0; 112.8	
HIV and ART status				<0.001
HIV−	58.8 (923)	98.4	96.2; 100.6	
HIV+/ART-naïve	36.1 (567)	115.6	112.7; 118.5	
HIV+/on ART	5.0 (79)	107.6	100.2; 114.3	
Body mass indexpercentile^2^				<0.0001
<25	41.7 (653)	108.7	106.4; 111.0	
25–50	51.5 (806)	103.4	99.1; 107.6	
50+	6.8 (107)	95.9	92.6; 99.2	
Serum C-reactive protein(mg/L)				<0.001
≤2	19.2 (300)	87.7	84.1; 91.4	
2–5	7.4 (115)	98.6	93.3; 103.8	
5–10	5.9 (92)	105.4	96.9; 113.9	
10–50	18.5 (289)	109.5	105.2; 113.7	
50+	49.1 (767)	110.8	108.4; 113.2	
Serum α_1_-acid glycoprotein(mg/L)				<0.001
≤1	25.0 (391)	86.8	84.0; 89.7	
1–2	20.2 (317)	109.1	105.0; 113.2	
2–3	38.1 (596)	111.1	108.4; 113.9	
3+	16.8 (263)	113.1	108.8; 117.4	

1 Pulmonary TB status was based on culture, except where culture data were not available. For 355 consecutively recruited sputum positive TB patients a control was randomly selected among individuals with same sex and age from the neighbourhood. Serum 25(OH)D data were available on 1570, but n may sum up to less, due to missing data.

2 Based on sex-specific percentiles among the neighbourhood controls.

The roles of sex, age, anthropometry, PTB and HIV and ART status, and elevated acute phase reactants as correlates of serum 25(OH)D, and interaction by TB, were assessed by multivariable analysis, with adjustment for year and quarter. Since there was no difference between PTB− and PTB+, these groups were combined as PTB. Without elevated serum acute phase reactants in the model (**Table 2** **, model 1**), sex, PTB and HIV and ART status were correlates of serum 25(OH)D, while age, BMI, AMA and AFA were not. However, there was a strong interaction between PTB and sex (P<0.0001), since PTB was associated with 24.8 (95% CI: 18.6; 30.9) nmol/L higher serum 25(OH)D compared to controls among females, and 9.8 (95% CI: 4.5; 15.2) nmol/L among males. With PTB accounted for in the model separately for females and males, females had 13.8 (95% CI: 8.2; 21.9) nmol/L lower serum 25(OH)D. ART-naïve HIV infected had 9.6 (95% CI: 4.5; 15.2) higher serum 25(OH)D compared to uninfected, whereas there was no difference between HIV infected on ART and HIV uninfected. The association between HIV and serum 25(OH)D was not different between controls and PTB (interaction p = 0.62). Elevated serum AGP was a strong positive correlate of serum 25(OH)D (Table 2, **model 2**), while elevated serum CRP was not. Adjustment for elevated serum AGP reduced the strength of association of serum 25(OH)D with PTB, but not HIV.

**Table 2:** Correlates of serum 25(OH)D in 1250 pulmonary TB patients and 355 neighbourhood with regression coefficient B, 95% confidence interval (CI) and P-values^1^.

	Model 1^2^			Model 2^3^		
	B	95% CI	P	B	95% CI	P
Sex						
Female	−13.8	−21.9; −8.2	<0.0001	−13.6	−20.4; −6.9	<0.0001
Male	–			–		
PTB status^1^						
PTB among females	24.8	18.6; 30.9	<0.0001	16.4	8.8; 24.0	<0.0001
PTB among males	9.8	4.5; 15.2	<0.0001	−0.7	−8.0; 6.6	0.83
Non-TB control	–					
HIV status						
HIV+/ART-naïve	9.6	6.0; 13.2	<0.0001	8.9	5.2; 12.5	<0.0001
HIV+/on ART	1.0	−6.6; 8.6	0.80			
HIV−	–			–		
Serum α_1_-acid glycoprotein (mg/L)						
<1				–		
1–2				11.8	5.5; 18.1	<0.0001
2–3				11.6	5.2; 18.0	<0.0001
3+				12.7	5.7; 19.7	<0.0001

1 Pulmonary TB status was based on culture, except where culture data were not available. For 355 consecutively recruited sputum positive TB patients a control was randomly selected among individuals with same sex and age from the neighbourhood. Age, and quarter and year of recruitment were adjusted for in both models.

2 Model 1: N = 1540, adjusted R2 = 0.206 and intercept = 112.1 (95% CI: 105.1; 119.2).

3 Model 2: N = 1537, adjusted R2 = 0.212 and intercept = 110.4 (95% CI: 103.3; 117.5).

While none of the anthropometric variables were associated with serum 25(OH)D with infections or serum AGP in the model, the role of these variables were assessed separately. Interestingly, interactions with respect to serum 25(OH)D were found between PTB and both AFA (p = 0.03) and BMI (p = 0.051), but not AMA (p = 0.47), due to negative associations among controls, but not PTB patients. For example, the regression coefficient was −1.2 (95% CI: −2.0; −0.3) for BMI among controls, but −0.1 (95% CI: −0.7; 0.5) among PTB patients.

## Discussion

We found that PTB patients had considerably higher serum 25(OH)D than non-TB controls, contrary to what has been reported from a number of other studies. A systematic review of studies published from 1980–2006 with data on serum 25(OH)D on PTB patients and controls was recently conducted [5]. Of seven case-control studies identified with a total of 531 participants, five studies reported lower serum 25(OH)D in cases compared to controls, while two studies did not. For all studies combined, serum 25(OH)D was 0.68 SD lower among cases. However, the sample sizes were small, ranging between 30–145 participants. Some studies did not use culture for diagnosing TB, some included extra-pulmonary TB, and selection of controls was not optimal. More recently, a study from Guinea-Bissau reported lower mean serum 25(OH)D in PTB patients compared to unmatched healthy controls, but a higher proportion of severe vitamin D deficiency (<25 nmol/L) in the controls [3]. A study from Greenland found a U-shaped relationship between serum 25(OH)D and PTB, in that both levels below 75 and above 140 nmol/L were associated with PTB [9].

Thus, the findings from case-control studies are inconsistent, which may be due to selection bias. It may also reflect limitations of the case-control design to assess the role of vitamin D status as a risk factor for TB. For example, data on vitamin D status are usually collected after TB disease has been diagnosed. It is therefore not possible to draw any inferences about the direction of a potential cause-effect relationship. TB disease itself may lead to changes in sun exposure and dietary and supplemental vitamin D intake, and such changes may be setting-specific. TB disease and treatment may also have direct biological effects on the metabolism of vitamin D. E.g., isoniazid and rifampicin has been reported to reduce serum 25(OH)D [22], so if vitamin D status is assessed after commencement of TB treatment, which is usually inevitable, this will create or contribute to an association between low serum 25(OH)D and TB. The acute phase response accompanying TB disease may also affect serum 25(OH)D independently of vitamin D status per se, and as such impair the validity of serum 25(OH)D as a marker of vitamin D status. This is likely, since 25(OH)D is bound to vitamin D binding protein (VDBP) and albumin in the blood, and many transport proteins, and indeed albumin, are negative acute phase reactants, ie declines during the acute phase response. Since several studies have not been able to demonstrate such an effect of the acute phase response, serum 25(OH)D has been considered to be a valid measure of vitamin D status, even during infections. For example, there was no evidence of a decline in a study among 14 children followed during a malaria attack leading to hospitalization [23]. Similarly, in our previous study among PTB patients in Mwanza, we failed to find any association between elevated serum levels of the acute phase reactant α_1_-antichymotrypsin and serum 25(OH)D [1]. However, two prospective studies among patients undergoing surgery found that serum 25(OH)D declined [10], [11]. The decline in serum 25(OH)D may not only be explained by the decline in serum concentrations of the transport protein, but could also be due to increased uptake by macrophages [24]. The partly transient decline in serum 25(OH)D seen in these studies may explain the previously found negative associations between serum 25(OH)D and TB.

However, we found that elevated serum AGP was associated with considerably higher serum 25(OH)D, and that this partially explained the higher serum 25(OH)D in PTB patients. AGP is a relatively slow reacting acute phase protein, so that high serum AGP reflects more long-lasting disease. In contrast, we found no association between serum CRP, a fast reacting acute phase protein, and serum 25(OH)D in a multivariable model. This is accordance with our previous finding of no association between serum α_1_-antichymotrypsin (ACT), another relatively fast-reacting acute phase protein, and serum 25(OH)D [1]. Interestingly, based on a study in infants, associations between serum AGP and other markers of inflammation, and levels of vitamin D binding protein have previously been reported [25]. Our observation that serum 25(OH)D was lower with delayed blood sampling is likely to reflect a rapid decline after start of TB treatment. Hence, since the acute phase response per se seem to reduce rather than increase serum 25(OH)D (), then this is not explained by the fast decline in acute phase reactants The decline is probably too rapid to be explained by sequestration of vitamin D in newly deposited fat tissue [26] and may instead be due to the effect of isoniazid and rifampicin as previously reported [22].

As for the relationship between serum 25(OH)D and PTB, the strength of our study is the random selection of age-sex-matched non-TB neighbourhood controls and the size of the study. There are several possible explanations for our finding of a higher serum 25(OH)D among PTB patients. First, greater sun exposure among PTB patients during diagnosis and treatment of PTB cannot be ruled out, whereas higher dietary or supplementary intake in response to PTB diagnosis seems unlikely in this population. Second, it could be due to reverse causality, ie that high vitamin D status is a risk factor for PTB, as recently hypothesised [9]. High vitamin D status has generally been associated with beneficial health effects, but several studies now suggest that high vitamin D status is associated with risk of disease or even all-cause mortality [27]. Third, and most plausible, vitamin D may be released from fat tissue lost during TB disease. Vitamin D is known to be sequestered in fat tissue, which explains the negative association between BMI and serum 25(OH)D found in studies among healthy individuals [26], [28], [29]. Indeed, several studies have reported considerable increments in serum 25(OH)D after surgically induced weight loss (26–28).

So, it is likely that while a mild acute phase response leads to a transient decline in serum 25(OH)D [10], [11], a more severe and sustained response leading to loss of fat mass results in an increase in serum 25(OH)D, although metabolism of 25(OH)D may also be affected. Two observations from our study seem to support this interpretation. We found, in accordance with other studies [26], a negative relationship between both BMI and AFA, but not AMA, and serum 25(OH)D among controls. Interestingly, this expected negative relationship was not seen among our PTB patients, neither in this nor in our previous study. This may suggest that the usual relation between serum 25(OH)D and measures of fat is disturbed in PTB patients, possibly due to release of vitamin D from fat tissue accompanied by changes in the metabolism of vitamin D and binding proteins. We also found a strong interaction between PTB and sex, as females had considerably lower serum 25(OH)D than males among controls, whereas there was no sex difference among PTB patients. It is known that females lose relatively more fat than males during PTB as well as HIV [30]–[33], and gain more during treatment (Praygod, submitted). So, the lack of sex difference among PTB patients could be due to greater loss of fat among females compared to males, hence a greater release of vitamin D.

We also found that HIV was associated with higher serum 25(OH)D, and this was seen both among PTB patients and non-TB controls. In contrast to PTB, the association with HIV was not explained by the acute phase response, as HIV infection per se does not lead to an acute phase response [34], or even impair the response to other stimuli [35].

### Year-to-year and Seasonal Variation

The seasonal variation, with highest serum 25(OH)D in the first half of the year, was in accordance with our study among PTB patients in 2001–2 [1]. In addition, this current study strongly suggests considerable year-to-year variation in vitamin D status. Not only was the mean serum 25(OH)D in the current study much higher compared to the previous study (111 vs 86.7 nmol/L), but in the current study serum 25(OH)D levels were higher in 2006–7 compared to 2008. The two studies were conducted in similar study populations of newly diagnosed pulmonary PTB patients, and the serum 25(OH)D analyses were done with similar analytical equipment at the same laboratory. Although our current data suggest that serum 25(OH)D increases during the acute phase response, the variation is not explained by changes in infectious disease burden, since we controlled for elevated serum AGP. We therefore believe that both seasonal and year-to-year variations reflect true variation in vitamin D status over time, due to variation in sun exposure and dietary intake of vitamin D.

### Conclusion

We found that PTB and HIV were associated with higher serum 25(OH)D. Elevated serum AGP was also a strong positive correlate of serum 25(OH)D, and explained of the association with PTB. We suggest that while S-25(OH)D may decline transiently during a mild acute phase response, it may increase if the acute phase response is severe and sustained and leads to loss of fat. Differences in treatment lag time, and hence magnitude of fat loss, may explain some of the inconsistencies between studies. The validity of S-25(OH)D as a marker of vitamin D status may be affected by infections. There is a need for prospective studies to assess the relationship between vitamin D and risk of TB, HIV and other infectious diseases.
